# Best interests versus resource allocation: could COVID-19 cloud decision-making for the cognitively impaired?

**DOI:** 10.1136/medethics-2020-106323

**Published:** 2020-05-06

**Authors:** Jordan A Parsons, Harleen Kaur Johal

**Affiliations:** Centre for Ethics in Medicine, Bristol Medical School, University of Bristol, Bristol, UK

**Keywords:** allocation of health care resources, capacity, clinical ethics, decision-making

## Abstract

The COVID-19 pandemic is putting the NHS under unprecedented pressure, requiring clinicians to make uncomfortable decisions they would not ordinarily face. These decisions revolve primarily around intensive care and whether a patient should undergo invasive ventilation. Certain vulnerable populations have featured in the media as falling victim to an increasingly utilitarian response to the pandemic—primarily those of advanced years or with serious existing health conditions. Another vulnerable population potentially at risk is those who lack the capacity to make their own care decisions. Owing to the pandemic, there are increased practical and normative challenges to following the requirements of the Mental Capacity Act 2005. Both capacity assessments and best interests decisions may prove more difficult in the current situation. This may create a more paternalistic situation in decisions about the care of the cognitively impaired which is at risk of taking on a utilitarian focus. We look to these issues and consider whether there is a risk of patients who lack capacity to make their own care decisions being short-changed.

## Introduction

The COVID-19 pandemic is putting the NHS under unprecedented pressure, requiring clinicians to make uncomfortable decisions they would not ordinarily face. These decisions revolve primarily around resource allocation in intensive care units (ICUs) and whether a patient should undergo invasive ventilation. Certain vulnerable populations have already featured in the media as falling victim to an increasingly utilitarian response to the pandemic. Those of advanced years or with serious existing health conditions are being deprioritised, and in some cases have had DNACPR notices applied pre-emptively and without consultation. A further vulnerable population potentially at risk of such treatment during the pandemic is those who lack the capacity to make their own care decisions.

Cognitively impaired patients have their rights protected by the Mental Capacity Act 2005 (MCA 2005). However, owing to the pandemic, there are increased practical and normative challenges to following the requirements of the Act. Capacity assessments to determine the need for best interests decisions, as well as the best interests decisions themselves, are likely to prove more difficult in current circumstances due to a variety of obstacles. This may create a more paternalistic situation in decisions about the care of the cognitively impaired, which is at risk of taking on a more utilitarian focus. We look to these issues and consider whether there is a risk of patients who lack capacity to make their own care decisions being short-changed. Further, we suggest that a lasting impact on the care of cognitively impaired patients is a realistic expectation. As a result of these concerns, we assert the need for clinicians to remain aware of the requirements of the MCA 2005 and continue to promote the rights of patients who lack decision-making capacity even during the unprecedented pressures of the pandemic.

## Capacity assessment practicalities

Delirium has been increasingly recognised as a presenting feature of COVID-19, particularly in patients of advanced age. This results in acute, fluctuating consciousness and temporary cognitive impairment.^1^ Patients who would ordinarily have the capacity to make decisions relating to their care and treatment may therefore require best interests decisions to be made on their behalf, as per the MCA 2005. In order to determine whether a patient lacks capacity to make these decisions, a full capacity assessment must be performed. This involves testing the patient’s ability to understand information relevant to a healthcare decision, retain it, and then weigh up the information before communicating their decision. Importantly, capacity is decision specific and a patient may lack capacity to make some decisions, but not others.^i^ As delirium has a fluctuating course, it is also feasible that a patient may transiently regain capacity to make decisions relating to their care and treatment. Hence, assessment of the patient’s capacity must remain under ongoing review. In the context of a pandemic, however, increasing pressures on doctors may impede on their ability to carry out capacity assessments in full.

The MCA 2005 states that a person should not be “treated as unable to make a decision unless all practicable steps to help him to do so have been taken without success”. This might involve a translator if a person prefers speaking in their native language, or engagement with specialist services such as speech and language therapy (SALT).^2^ Often, it takes time to arrange these additional services. It is also possible that these services will be stretched due to staff sickness or increased demand. For instance, SALT services may be required to support increasing numbers of patients in regaining laryngeal function following intubation and ventilation.^3^ Doctors may not, for these reasons, be able to take ‘all practicable steps’ as they usually would. It is also likely that their own ability to communicate with patients will be impaired, and the importance of both verbal and non-verbal communication has long been emphasised.^4^ Although the use of personal protective equipment (PPE) is necessary to reduce cross-infection, wearing masks and eyewear may further disorientate a confused patient in an unfamiliar environment. The use of PPE may also be challenging for patients who, for example, are hard-of-hearing and reliant on their ability to lip-read. There is, then, reason to be concerned that capacitous patients may be identified as lacking capacity following suboptimal capacity assessments, and best interests decisions may be made inappropriately on their behalf. This would undermine such patients’ autonomy.

## Best interests practicalities

Under the MCA 2005, patients who lack capacity to make decisions about their own care must have the decision made in their best interests, and the decision-maker would usually be the doctor providing treatment and care.^ii^ The decision-making process requires that doctor to consult with those who know the patient well, which usually means close relatives. Any decision made must not be based on the fact that the patient lacks decision-making capacity.^2^ If a patient is deemed to lack capacity and the requirements of the MCA 2005 become applicable, a new set of obstacles present themselves. Amidst the pressure of the pandemic, the process of making a best interests decision is complicated.

One issue is staff redeployments. Clinicians from various fields are being moved to ICUs and required to work outside of their usual remit. ICUs may currently be leaving specialist care decisions to those from relevant specialties with the necessary expertise, but it is possible that during the peak of the pandemic the finite number of such specialists will be insufficient to do so. There are also concerns that this shortage may be worsened by late presentations not related to COVID-19. Seeking not to overwhelm the NHS, patients are choosing not to seek medical attention when they otherwise would have, which is reflected in the 29.4% decrease in the total number of attendances to accident and emergency in March 2020 as compared with the same time last year.^5^ This is resulting in later presentations which require more medical attention. For example, a patient with a heart attack may not seek medical attention and later present with heart failure, requiring the attention of clinicians with specialist knowledge.^6^ This additional strain on senior staff from relevant specialties may necessitate decision-making by less senior staff, potentially from other specialties. If such staff have been redeployed from areas where best interests decisions are uncommon, they may lack the experience to ensure these decisions are made properly. This could result in decisions being made which are not in the best interests of patients.

Further difficulties arise in arranging a best interests meeting. Where decisions are time sensitive, the demands on staff already outlined may result in fewer members of the care team being able to attend. This may affect outcomes by losing a potentially valuable insight, particularly if nurses who have been involved in the care of a particular patient are unable to attend. Nurses typically spend more time with patients and may have a greater understanding of their values and preferences than the patients’ doctors. As a result, they are often able to provide doctors with information about the patient and act as advocates.^7^ Therefore, the inability of nurses to fully contribute to these decisions risks diminishing the role of the patient’s own values and preferences in decisions about their care. It is also possible that nurses will not have the same level of familiarity with patients they normally would due to redeployments and covering staff shortages, which further erodes their ability to contribute to best interests decisions.

Of course, the primary intended source of information pertaining to what the patient would have wanted is consultees. However, here rests another challenge to the practice of best interests during the pandemic. The risk of infection has resulted in most hospitals stopping or significantly limiting patient visiting. Consulting those close to patients when making best interests decisions, then, will have to be done remotely. Some trusts are introducing virtual visits for patients who do not have suitable devices of their own,^8^ but this might still limit the ability of consultees to communicate with the patient in order to best represent their preferences. If a patient communicates non-verbally, relatives are often best placed to communicate with them as they are more aware of how to interpret visual cues. For example, in a recent case before the England and Wales Court of Protection (EWCOP)—which we will soon discuss—the patient’s mother disagreed with the Trust about her daughter’s limited ability to communicate, and noted how her daughter became animated at the suggestion that she was going to challenge the Trust.^9^ Such communication may not be possible through video call, which could affect the role of the consultee in decisions and result in the patient’s perspective being lost.

## Best interests versus resource allocation

Resource allocation is the issue receiving the most attention in the bioethics space at present. This focus is necessary, but where it infringes on ethical values it is just as necessary to question the ethical robustness of approaches. Side-lining the best interests of cognitively impaired patients is not appropriate, and care should be taken to avoid this.

Efforts to better allocate scarce resources have already led to unpalatable decisions. A GP surgery in Wales recently wrote to a number of its patients with significant illnesses asking them to complete DNACPR forms, explaining to them that this would allow scarce ambulance resources to “be targeted to the young and fit who have a greater chance”.^10^ Residents of several care homes in both Wales and East Sussex have also had DNACPR notices applied to their care plans as a blanket policy without having been consulted.^11^ Both of these decisions have been criticised as inappropriate, and they clearly show that the relative value of the more vulnerable members of society is being brought into question.

As tactless as they may have been, the main factors driving many of these moves were justified, namely comorbidities. This is reflected in the National Institute for Health and Care Excellence’s (NICE) COVID-19 guidance on critical care for adults which clarifies that comorbidities and underlying health conditions ought to be considered in all cases.^12^ While the GP surgery and care home examples were not directly best interests decisions, it is probable that at least some of the patients and residents would have lacked capacity to consent to a DNACPR notice. It is also worth noting that NICE recently faced heavy criticism, and were threatened with judicial review^13^ for advocating the use of the Clinical Frailty Scale (CFS) to determine the suitability of all adults for hospital treatment in their initial COVID-19 guideline for critical care.iii Through this scoring system, a person unable to carry out high-order, independent activities of daily living would be scored ‘5’, thereby classifying them as ‘mildly frail’. The NICE guideline suggested that it may not be appropriate to provide patients with a score of 5 or more with hospital treatment. This classification was thought to unfairly discriminate against individuals with stable cognitive impairment, such as those with learning disabilities or autism. The CFS has since been declared as unsuitable for assessing frailty in patients with learning disabilities or under the age of 65. Instead, an individualised approach to assessing escalation of treatment has been deemed necessary, in consultation with family and/or paid carers.^14^ However, the utilisation of the CFS in the initial guideline demonstrates that the pressure to develop rapid national guidance has resulted in considerations of its application being overlooked, particularly in relation to vulnerable populations.

A recent case in the EWCOP, *University Hospitals Bristol NHS Foundation Trust v ED*,^9^ sought declarations that it would be lawful for ED’s treatment not to be escalated if her condition deteriorated. ED’s doctors opposed escalation in the form of CPR or ICU admission on the basis that they do not consider it in the patient’s best interests, with Mr Justice Moor noting in his judgement that one “says that this is not about rationing ICU beds. It is a best interests decision that he and Dr DF are agreed upon”. However, it ought to be questioned what role resource allocation may have played in this decision.

ED had previously been admitted to the ICU on several occasions and had three tracheostomies. On these occasions, no declaration from the EWCOP was sought. It was during a pandemic that is putting pressure on ICUs that the patient’s doctors felt it would be in ED’s best interests not to receive such care. That is not to say that the decision of the doctors was not made in the best interests of the patient, but it is possible that it was at least slightly influenced by pandemic considerations. Extrapolated, this could be interpreted in three ways:

1. The pandemic is prompting doctors to think more about rationing, which may result in the devaluing of the lives of patients who lack decision-making capacity and decisions not being made in their best interests (if one believes the doctors in this case were incorrect);
2. The pandemic is prompting doctors to think more about advance care planning (ACP), but merely as a practical consideration with no view to the pressures of the pandemic affecting the care a patient will or will not receive;^iv^ or
3. The pandemic is prompting doctors to think more about rationing, which may result in patients who lack decision-making capacity having decisions made which *happen to be* more so in their best interests (if one believes the doctors in this case were correct).

It is possible, and indeed most likely, that all three are happening as a result of the pandemic. However, the second interpretation is the only one that is ethically reconcilable. Interpretations (1) and (3) are allowing the pandemic to affect the care of patients who lack decision-making capacity. Even if it is affected for the better—meaning interpretation (3) resulting in decisions being made in the best (better?) interests of the patient—it is still an instance of a preoccupation with resource allocation affecting care, which is concerning. Interpretation (2), however, is neutral in this regard and is therefore ethically reconcilable. An ACP would allow a patient who has decision-making capacity to take a more active part in decisions about their care when first admitted to hospital, and prepares for the likely event they lose that capacity.^15^ Assuming there is no attempt to persuade a patient in a particular direction, this would both enhance the autonomy of the patient before their situation deteriorates and relieve some pressure on clinical staff at a critical time for the NHS.

Yet there is evidence of how the pandemic has already compromised best interests decisions in social care. In order to increase bed capacity in acute NHS hospitals, the Coronavirus Act 2020 established that there is no longer a legal duty to provide NHS Continuing Healthcare (CHC) assessments to determine the care and residential needs of patients who are medically fit for discharge from hospital. Without these assessments, patients may be discharged into an environment over which they have little choice. While hospitals will have a duty to ensure they are discharged into a safe environment which meets their care needs (for example, home with a care package or a community hospital), patients have temporarily lost access to the mechanism through which they can explore their care or residence options free of charge. This emergency legislation has been justified in the context of the pandemic, serving the greater purpose of freeing up acute hospital beds. However, the impact of this on patients who lack the capacity to make decisions on their discharge is particularly concerning. As Ruck-Keene notes, there is considerable overlap between patients with impaired decision-making capacity and continuing healthcare needs, and their discharge options have been significantly narrowed by this legislative change.^16^ Rather than a multidisciplinary exploration of the values and preferences of the patient, in which the views of consultees are taken into account, a decision will be made by the patient’s medical team to ensure the patient’s safety and facilitate a quick discharge. Though these patients will have access to CHC assessments following the emergency period, there will invariably be a backlog creating further delays in determining what care and residential support is in the patient’s best interests.

The demands of the pandemic have affected the ability of the NHS to provide care in general. As we hit the peak, it is unlikely that all patients will be able to receive life-saving care and yet more difficult decisions will have to be made. For instance, decisions about the discontinuation of the treatment of patients already receiving intensive care will have to be made. In such situations, Wilkinson reinforces the equivalence thesis in noting no difference between withholding and withdrawing treatment ceteris paribus.^17^ When this point is reached, cognitively impaired patients ought not to receive special treatment, but also not lesser treatment on the basis of their impairment. Doctors making decisions about the continuation of intensive care for cognitively impaired patients during the pandemic should be especially conscious of any resource allocation influence.

## Lasting impact?

Decisions being made in the NHS during the pandemic are being made very much in the context of the pandemic. The situation is, arguably, necessitating a utilitarian outlook to respond to the questions of resource allocation.^18^ One would hope, therefore, that things would return to ‘normal’ afterwards. Is this a realistic expectation, though?

If a decision is made which is not in the best interests of a patient during the pandemic, and this is not a single occurrence, it begins to devalue the lives of patients who cannot make their own care decisions. Not providing care on a utilitarian basis, factoring in the patient’s cognitive impairment, is against the principles of the MCA 2005. Indeed, it begins to erode the rights of these patients under the United Nations Convention on the Rights of Persons with Disabilities.^19^

What seems more realistic, then, is that this will have a lasting impact. That is not to say that there will be a prevailing assumption that patients who lack decision-making capacity ought not to receive intensive care. Such a situation has not arisen amidst the pandemic and is unlikely to. Rather, the basis that a cognitively impaired patient ought not to have their care affected because of that impairment may be undermined. There could be a mirroring of decisions made during the pandemic outside of emergency situations in the future, with doctors questioning more the value of invasive procedures to preserve the life of such patients.

## Conclusion

There is no doubt that the COVID-19 pandemic is necessitating difficult decisions, and expecting any part of the NHS to operate as it usually does is unrealistic. Nonetheless, there are certain values which should not be easily side-lined, including the protection of the right of patients lacking decision-making capacity to have decisions about their care made in their best interests. Early responses to the pandemic have demonstrated a devaluing of the more vulnerable in society. Coupled with the practical difficulties facing the best interests process, we suggest that there is a very real risk of decisions being made which are not in the best interests of cognitively impaired patients—especially if such patients become infected with the virus and require invasive respiratory support.

This is not an assault on doctors. Doctors are under immense pressure handling the pandemic and have spoken out about how uncomfortable they are with some of the decisions they are being forced to make, especially given the lack of national guidance to ensure consistency in the decision-making process. However, it is important for them to uphold the principles of the MCA 2005 even under this pressure. In particular, doctors must consider the reasons behind decisions they are making about the care of patients who lack decision-making capacity—are they being made in the best interests of that patient, or are other factors influencing them? Of course, doctors retain a right not to provide care that they consider futile or harmful, but this must be carefully navigated. Further, supporting patients in making decisions where possible and the involvement of consultees in decisions remains both a requirement under the MCA 2005 and an ethical imperative, so efforts must be made to minimise the impact of practical barriers on broad involvement in treatment decisions.

Efforts to uphold the principles of the MCA 2005 during the pandemic and not devalue the lives of those who cannot make decisions about their own care will also help to minimise the risk of COVID-19 having a lasting, negative impact on the rights of cognitively impaired patients. Undoubtedly, there will be lasting changes to the NHS following the pandemic, but it is important to reduce the likelihood of ethically problematic lasting changes. To protect the rights of the cognitively impaired at a time like this is necessary to ensure that they are not brought into question when life begins to return to normal.

## Footnotes

For readability, any reference to a patient’s lack of decision-making capacity relates solely to the care decision being discussed.

Assuming there is no legally appointed proxy decision-maker.

The original guidelines have since been updated.

This interpretation might only be considered more broadly as it is unlikely to apply to the case discussed. In the case, the patient is cognitively impaired to the extent that she is unlikely to be able to meaningfully participate in ACP.
